# Spatio-temporal analysis of rice production and trade between Southwest China and major rice producers in Southeast Asia

**DOI:** 10.3389/fpls.2025.1543314

**Published:** 2025-03-21

**Authors:** Yucong Geng, Qurat-Ul-Ain Raza, Muhammad Amjad Bashir, Shufu Xie, Xuemei Song, Mingyue Yan, Guodong Jia, Ruotong Liu, Wangfang Ran, Yupei Long, Mengyuan Zhou, Abdur Rehim, Xinjian Liang

**Affiliations:** ^1^ Center for Southeast Asian Economic and Culture Studies, Chengdu Normal University, Chengdu, China; ^2^ Sichuan Provincial Key Laboratory of Philosophy and Social Sciences for Monitoring and Evaluation of Rural Land Utilization, Chengdu Normal University, Chengdu, China; ^3^ Department of Soil Science, Faculty of Agricultural Sciences and Technology, Bahauddin Zakariya University, Multan, Pakistan; ^4^ Department of the History of Science, Tsinghua University, Beijing, China

**Keywords:** rice trade, international trade, food security, spatial analysis, market share

## Abstract

Analyzing the spatial and temporal patterns in rice production and trade in the southwestern region of China and the main rice-producing countries of Southeast Asia provides a reference for trade development and food security. The study aims to analyze the production costs and rice import and export volumes between Southwest China and the main rice-producing countries in Southeast Asia. For the current study, rice sown area, yield, production, and import/export data were collected for 1978-2021 based on the FAO statistical database. Comparative analyses of the planted area, unit area production, total production, import and export, market share, trade potential, and trade competency of rice in China’s southwestern region and the major rice-producing countries in Southeast Asia were performed. The study revealed that rice cultivated area and total production in the major rice-producing countries of Southeast Asia generally increased from 1978 to 2021, with Indonesia, Vietnam, and Thailand being among the top three major rice-producing countries in Southeast Asia since 1978. Cambodia’s area under rice cultivation has increased significantly, with its total rice production reaching from 1 million tonnes in 1978 to 11.41 million tonnes in 2021. The import of rice in the southwestern region of China is increasing, and the volume of rice trade in 1978 was nearly 12 times higher than that in 2021. In addition, Thailand and Vietnam are the major rice exporting countries, accounting for 53.49% and 35.10% of the rice export volume, and they can be used as potential countries for future rice trade with the southwestern region of China. In conclusion, increasing agricultural inputs to ensure regional food security, deepening rice industry chain reform, expanding the rice trade market in Southeast Asia, and establishing a high-standard free trade zone to smooth regional trade in rice can strengthen trade exchanges and foster new trade growth.

## 1 Introduction

Considering the fertility rate, the global population is projected to increase by 10% in 2030 and reach 8.5 billion, 26% in 2050 and 9.7 billion, and 42% in 2100 and 10.9 billion, respectively. It can also cross 17 billion in 2100 if these fertility and mortality rates remain constant (Bin Rahman and Zhang, 2023). The increase in population comes with major threats including resource repletion (Ganivet, 2020), food security, environmental degradation (Ukaogo et al., 2020), and economic constrains (Bloom and Canning, 2006). Food security is a major concern for all countries worldwide in order to eliminate poverty. According to the World Food Summit of 1996, food security is achieved when all people have enough, safe, and nutritious food at all times to live an active and healthy life (Bandumula, 2018). Rice is an important food crop because almost half the global population consumes it daily, and it is considered a staple food. In addition, it supplies about 20% of the dietary energy of the world, followed by wheat (19%) and maize (5%) (Bin Rahman and Zhang, 2023).

Ensuring food security and enhancing agricultural productivity is the major challenge for the global economy, but rapid climate change and associated abiotic factors, including drought, rainfall, flooding, and temperature fluctuations, have adversely affected rice production. Climate change is affecting the agricultural productivity specifically in southeast Asia and western Asia (Ozdemir, 2022). During the next century, it is estimated that rice production will be reduced by 51% due to global climate change (Hussain et al., 2020) because crop production declines due to increased heat stress, reduced photosynthetic capacity, increased rice water requirements, and improved respiratory processes (Pickson et al., 2022).

Asia is the dominant global rice supplier, producing about 90% of the rice, and it is an equally important food crop for Asia and the world. In East and Southeast Asia, rice production was 418.56 million tonnes, covering 55.4% of global rice production in 2019 (Lin et al., 2022). Moreover, China is the largest rice-producing country, with 28% of the global rice production and a prime source of nutrition for nearly 65% of the Chinese population (Pickson et al., 2022). Southeast Asia has increased rice production over the last 50 years by enhancing rice yield and cropping intensity. In addition, the limited scope for main rice-producing countries like China and India to produce surplus rice and the continuous increase in rice trade has enhanced the opportunity for Southeast Asia to contribute to the global rice supply (Yuan et al., 2022).

Furthermore, the land per capita is 50% less than the world average, and the available water per person is one-third of China’s population because the country has limited resources to feed its vast population. In addition, extended urbanization and industrialization have also led to huge stress on agriculture (Zhan, 2022). It has also been projected that China’s water (50.5%) and land (10.5%) resources will be saved in 2030 (in comparison with 2015) with the increasing food imports in China (Huang et al., 2017). Asian countries such as India, Vietnam, and Thailand dominate the global rice trade; to understand the global market and rice production and consumption patterns for food security, it is crucial to study these countries (Yuan et al., 2022).

China introduced the One Belt and One Road (OBOR) policy to promote global economic integration, reduce trade costs, build an image as a global power, ensure peaceful orientation, and achieve sustainable development goals (Bashir et al., 2021). In addition, China has also made efforts to improve its relationship with Southeast Asia through The Belt and Road Initiative (BRI), which has built stable and friendly relations and promoted good economic partnership (Gong, 2019).

This paper aims to analyze the latest data collected on production costs, trade prices, and import and export volumes of rice between China and the main rice-producing countries in Southeast Asia. A comprehensive evaluation of the development trend of cooperation in rice trade in the international market and a specific analysis of its impact factors. Finally, we propose measures and methods to develop rice trade between Southwest China and Southeast Asia to improve rice’s trade and cooperation capacity in Southwest China. At the same time, combining the economic environment of domestic and foreign rice production and trade in Southwest China and Southeast Asia, analyzing the current situation of rice’s international competitiveness and the factors affecting its global competitiveness is of great practical significance for the development of agriculture in China and Southeast Asia, as well as for the long-term economic development of the inter-regional area. In addition, the spatial and temporal characteristics of rice production and evaluating the current level of rice production, supply, and demand has not been reported yet. Accurately predict the future rice industry and trade trends and provide theoretical support for the construction of policy guidance recommendations for developing the rice industry in China and future trade cooperation with Southeast Asian rice-producing countries. The objectives of the study are to (1) identify trade trends between Southwest China and major rice-producing countries in Southeast Asia (2) assess the major factors on rice production and trade, and (3) make recommendations to improve trade policies, and regional cooperation to ensure food security and economic stability.

## 2 Data sources and research methods

### 2.1 Overview of the research area

The southwest region of China mainly consists of three provinces: Sichuan, Guizhou, Yunnan, Chongqing (a direct-administered municipality), and Tibet (an autonomous region) - a total area of 2,340,600 km^2^, comprising 24.5% of China’s total land. The regional geographic location is between 97°21′E and 110°11′E, 21°08′N and 33°41′N, neighboring Myanmar, Laos and Vietnam.

**Figure 1:**
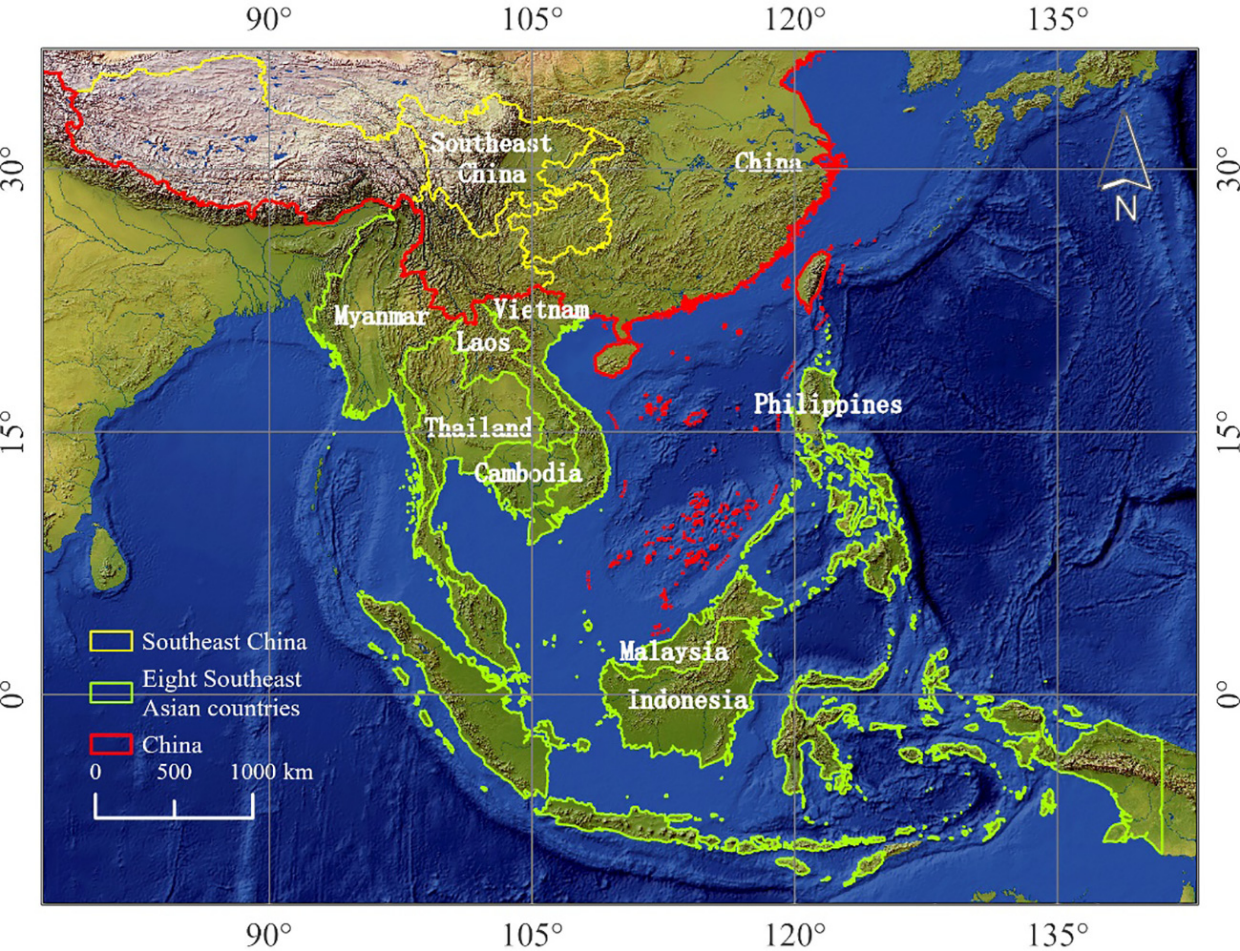
Distribution map of the main rice-producing countries in Southwest China and Southeast Asia.

The major rice-producing countries in Southeast Asia include Malaysia, Myanmar, the Philippines, Thailand, Indonesia, Vietnam, Cambodia, and Laos (**Figure 1**). The regional geographic location is 92°E to 140°E, 10°S to 28°26′N. Myanmar, Laos, Thailand, Vietnam, and Cambodia are located in the Central and Southern Peninsula, with a terrain characterized by mountains and rivers, a longitudinal distribution, a high terrain in the north and a low terrain in the south, and a predominantly tropical monsoon climate, with an abundance of annual precipitation, an evident drought and rainy seasons, and high temperatures throughout the year with an average annual temperature of more than 20°C. Indonesia, Malaysia, and the Philippines are in the Malay Archipelago region, with rugged, mountainous terrain and tropical rainforest climate, high temperatures and heavy rainfall throughout the year, and an average annual temperature of around 28°C.

### 2.2 Data sources

The southwestern part of China and the main rice-producing countries in Southeast Asia were selected for this study. The main statistics were obtained from the China Statistical Yearbook (https://www.yearbookchina.com/navisearch-2-0-0-1-china-0.html) and the Food and Agriculture Organization of the United Nations (FAO) statistical database FAOSTAT (http://www.fao.org/faostat/en/), focusing on the data on rice production and imports and exports between the major Southeast Asian countries and the southwestern region of China from 1978 to 2021. In 1978, when China was in the early stages of reform and opening, rice production developed rapidly. Data on trade in agricultural products between China’s southwestern region and the main rice-producing countries in Southeast Asia were obtained from the United Nations COMTRADE trade database, including trade data for 2011-2021. The import and export volumes and values of the relevant regions and countries for 2011-2021 were also collected. The data were selected from 2011 onwards, mainly because the main rice-producing countries in Southeast Asia, such as Laos, Cambodia, and the Philippines, had missing data on rice trade prior to 2011, making it impossible to compare them with other main rice-producing countries in Southeast Asia. Therefore, the data have been selected from 2011 onwards. Data on GDP was collected from the World Bank database. Data on proximity to the country and geographic distance was collected from the CEPII GeoDist database.

### 2.3 Research methods

#### 2.3.1 Linear model

Changing patterns of total rice production and planted area over time in Southwest China and Southeast Asian rice-producing countries from 1978 to 2021 were analyzed by one-dimensional linear regression models. The formula is as follows (Equation 1) (Chen et al., 2012; Al Mamun et al., 2021):


(1) $$Y_{I}=a+bx$$

Where $Y_{I}$ denotes the cultivated area (yield, total production) of the ith region, x is the year, a is a constant term, and b represents the coefficient of the cultivated area of rice concerning the year. The Pearson linear model was used to check the significance of rice cultivated area and year.

#### 2.3.2 Rate of contribution to production

This indicator reflects the dominant factors affecting rice yield, with the main influencing factors including the contribution of yields, the contribution of area, and the interaction between the two in three aspects. The formula for calculating the yield contribution of rice is as follows (Equation 2–4):


(2) $$RA_{ij}=(A_{j}-A_{i})\times \frac{Y_{i}}{P_{j}-P_{i}}$$

(3) $$RY_{ij}=(Y_{j}-Y_{i})\times \frac{A_{i}}{P_{j}-P_{i}}$$

(4) $$RR_{ij}=\frac{RY_{ij}}{RA_{ij}}$$

where i is the beginning year, and j is the end year, $RA_{ij}$ denotes the contribution rate of the sown area of rice in the time period ij , $RY_{ij}$ denotes the contribution rate of rice yield in the time period ij , $RR_{ij}$ denotes the contribution rate of yield. $A_{i}$ and $A_{j}$ denote the harvested area of rice, $Y_{i}$ and $Y_{j}$ denote rice yield, and $P_{i}$ and $P_{j}$ denote total rice production in time periods i and  j , respectively. Yield contribution rates by type are given in **Table 1**, which indicates if the index value is below 0.5 it falls under area-led yield contribution, an index value between 0.5 – 2 shows the mutual dominance of area and yield, and an index value greater than 2 indicates yield-led contribution.

**Table 1:** Breakdown of yield contribution rates by type.

Yield contribution index	Type of yield contribution
$RR_{ij}$ ≤ 0.5	Area-led
0.5 < $RR_{ij}$ < 2	the area and yield mutual domination type
$RR_{ij}$ ≥ 2	Yield-led

Here, $RY_{ij}$ denotes the contribution rate of rice yield.

#### 2.3.3 Indices of comparative advantage of scale and efficiency

In this study, the comparative advantage index of scale reflects the ratio of the difference in rice cultivating area between the southwestern region of China and the main rice-producing countries in Southeast Asia, and the comparative advantage index of efficiency reflects the ratio of the difference in rice yields between the two areas. Classifying 1978-2000 (22 years) as the first phase and 2001-2021 (21 years) as the second phase, the analysis focuses on the production of China and 11 other major rice-producing countries. The formulas for the comparative advantage of the size index and the comparative advantage of the efficiency index are given below (Equation 5, 6) (Yu et al., 2006):


(5) $$SAI_{i}=\frac{A_{i}\;-\;A}{A}$$

(6) $$EAI_{i}=\frac{Y_{i}\;-\;Y}{Y}$$

where $SAI_{i}$ and $EAI_{i}$ denote the indices of comparative advantage of scale and comparative advantage of efficiency in rice production, respectively; $A_{i}$ and $Y_{i}$ denote the rice cultivating area and the unit yield of rice in the main rice-producing countries of Southeast Asia, respectively; A and Y denote the cultivating area and rice yield in the southwestern region of China. When both $SAI_{i}$ and $EAI_{i}$ are less than 0, it means that the production scale and production efficiency of the main rice-producing countries in Southeast Asia are at a disadvantage compared with those in the southwest region of China; when both $SAI_{i}$ and $EAI_{i}$ are greater than 0, it means that the production scale and production efficiency of the main rice-producing countries in Southeast Asia are at an advantage compared with those in the southwest region of China.

#### 2.3.4 Comparative market share analysis

This indicator reflects the position of southwestern rice in the export markets of the main rice-producing countries in Southeast Asia. Using the proportion of Southwest China’s rice exports to the exports of major rice-producing countries in Southeast Asia as a measure, the value is between 0 and 1, the larger the relative market share, the stronger the security of the industry. The formula for its calculation is as follows (Equation 7) (Elewa, 2019):


(7) $$\begin{array}{c} \text{Market share}\;=(\text{Southwest China rice exports}/\\ \text{total rice exports from major rice producing countries in Southeast Asia)} \end{array}$$

#### 2.3.5 Net rice imports

Measuring import and export trade between China’s southwestern region and the main rice-producing countries of Southeast Asia in terms of net rice imports. The formula for calculating net imports of rice is as follows (Equation 8):


(8) Net rice imports=rice imports-rice exports

#### 2.3.6 Trade competitiveness

Trade competitiveness is the strength of the competitive advantage of rice exports from the southwestern part of China over the main rice-producing countries in Southeast Asia. The trade competitiveness of rice is calculated as follows (Equation 9) (Wang, 2023):


(9) $$TC=\frac{E-I}{E+I}$$

In the formula, TC indicates trade competitiveness; E and I indicate rice exports and imports in China’s Southwest or Southeast Asian rice-producing countries. The values are between - 1 and 1, the greater the trade competitiveness, the higher the degree of industrial security.

#### 2.3.7 Gravity modeling

With its growing popularity and deepening in the analysis of international trade, many multivariate variables that can affect the size of trade have gained prominence. We refined the model variables to analyze the relevant factors affecting rice trade between China’s southwestern region and the main rice-producing countries in Southeast Asia. Expanding on the base model, the following model (He, 2024; Leitão, 2024) was obtained (Equation 10):


(10) $$\begin{array}{c} lnimport_{ijt}=a_{0}+a_{1}lndist_{ij}+a_{2}S_{j}+a_{3}lnGDP\_per_{it}\\ +a_{4}regulation_{ijt}+a_{5}GDP_{jt}+{\text{ϵ}}_{\text{ijt}} \end{array}$$

In the formula, i represents China’s southwest region, j represents the main rice-producing counties in Southeast Asia, and $lnimport_{ijt}$ represent the amount of rice imports from China’s southwest region and the main rice-producing countries in Southeast Asia in time t . $lndist_{ij}$ means distance; when the distance is closer, the transport cost is lower, which is more favorable to rice imports. $S_{j}$ denotes crop sown areas in major rice producing countries in Southeast Asia. $lnGDP\_per_{it}$ denotes the GDP per capita of the Southwest region, which represents the income level of the Southwest region. $regulation_{ijt}$ denotes the distance between the economic systems of the major rice-producing countries in Southeast Asia and the Southwest region. $GDP_{jt}$ is the gross domestic product of the major rice-producing countries in Southeast Asia. ${\text{ϵ}}_{\text{ijt}}$ denotes the random error term. Refer to **Table 2** for the meaning and expected sign of the variables in the model.

**Table 2:** Meanings and expected impacts of variables in the gravity model of rice trade between China’s southwestern region and Southeast Asia’s main rice-producing countries.

Variant	Mean	Expected impact
$lndist_{ij}$	Geographical distance between the south-western part of China and the main rice-producing countries in South-East Asia	−
$S_{j}$	Crop harvested area in major rice-producing countries in South-East Asia	+
$lnGDP\_per_{it}$	GDP per capita in the south-west	+
$regulation_{ijt}$	Indicates the distance of the economic system between the south-western part of the country and the main rice-producing countries in South-East Asia	−
$GDP_{ijt}$	Gross domestic product of major rice-producing countries in South-East Asia	−

“+” denotes a positive impact; “-” denotes a negative impact.

Estimating the trade potential of rice imports is a matter of measuring the constructed trade gravity model. Comparison of the actual volume of trade T between the two economies with the modeled estimated trade volume $T^{\prime }$ . The specific formula is: $TP_{ij}=T$ / $T^{\prime }$ . Trade potential is classified into three categories based on the ratio: large potential, pioneering potential, and re-modeling potential (**Table 3**).

**Table 3:** Classification of trade potential.

Trade Potential Index	Type of trade potential
$TP_{ij}$ ≤ 0.8	High Potential
0.8 < $TP_{ij}$ < 1.2	Potential Exploitation
$TP_{ij}$ ≥ 1.2	Potential re-modelling

### 2.4 Statistical analysis

Data was collected and statistically analyzed using Microsoft Excel 2021^®^. ArcGIS Pro^®^ was used to complete the distribution and annual phase change maps. The Stata^®^ 15.1 was used to calculate the direction and magnitude of the impact of each influencing factor on trade volumes and to derive the trade value estimation equation, comparing the calculated value with the actual trade and analyzing the trade potential value.

## 3 Results

### 3.1 Comparative analysis

#### 3.1.1 Comparative analysis for the sown area

**Figure 2:**
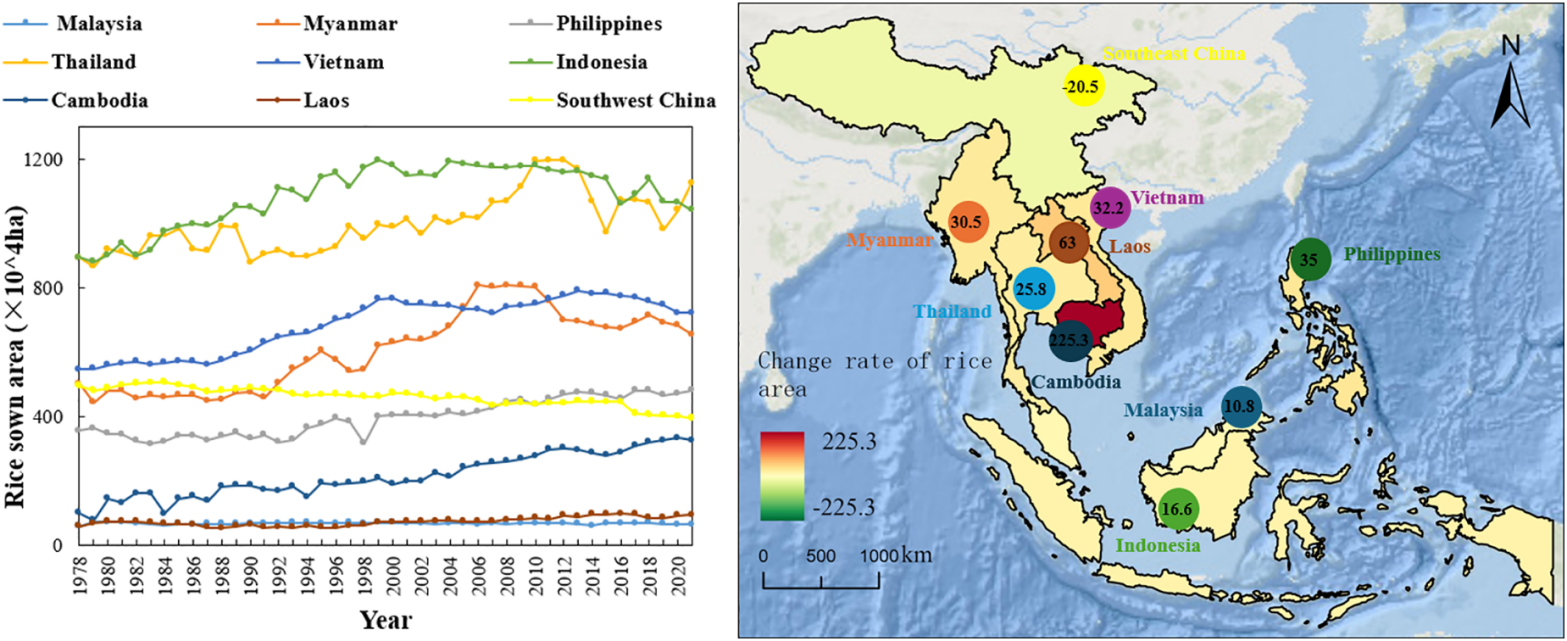
Change rate of rice sown area in southwest China and Southeast Asia.

The cultivating area of the main rice producing countries in Southeast Asia shows an overall increasing trend from 1978 to 2021 (**Figure 2**). The most significant increase is in Cambodia, from 1 million ha in 1978 to 3.25 million ha in 2021, a nearly 2.25-fold increase. The annual average rice growing area in Indonesia is 10,846,500 ha, which is 110.38% higher than the average annual area sown in the main rice-producing countries of Southeast Asia. Thailand’s rice growing area averaged 9,976,500 ha per year, higher than 93.50% of the average annual sown area of the main rice-producing countries in Southeast Asia. The area sown to rice in the two countries is significantly higher than in the other countries and regions; the area sown to rice in Vietnam, Myanmar and the Philippines is growing steadily. The average annual area sown to rice in the Lao People’s Democratic Republic and Malaysia has remained stable at between 14.21% and 13.07% that of the major rice-producing countries in Southeast Asia. Meanwhile, the rice growing area in the southwest China in 2021 was 3,942,700 ha, a decrease of 20.5% compared to 4,959,200 ha in 1978.

In summary, the rapid growth in the cultivating area of rice in Cambodia is the main driver of the expansion of rice production in Cambodia. Compared with other countries that have maintained a positive growth trend, the southwestern region of China has generally maintained a declining trend, with an average annual decline of 23,600 ha, indicating that it is more difficult to stabilize the area under rice cultivation in the southwestern region of China.

#### 3.1.2 Comparative analysis on rice yield

**Figure 3:**
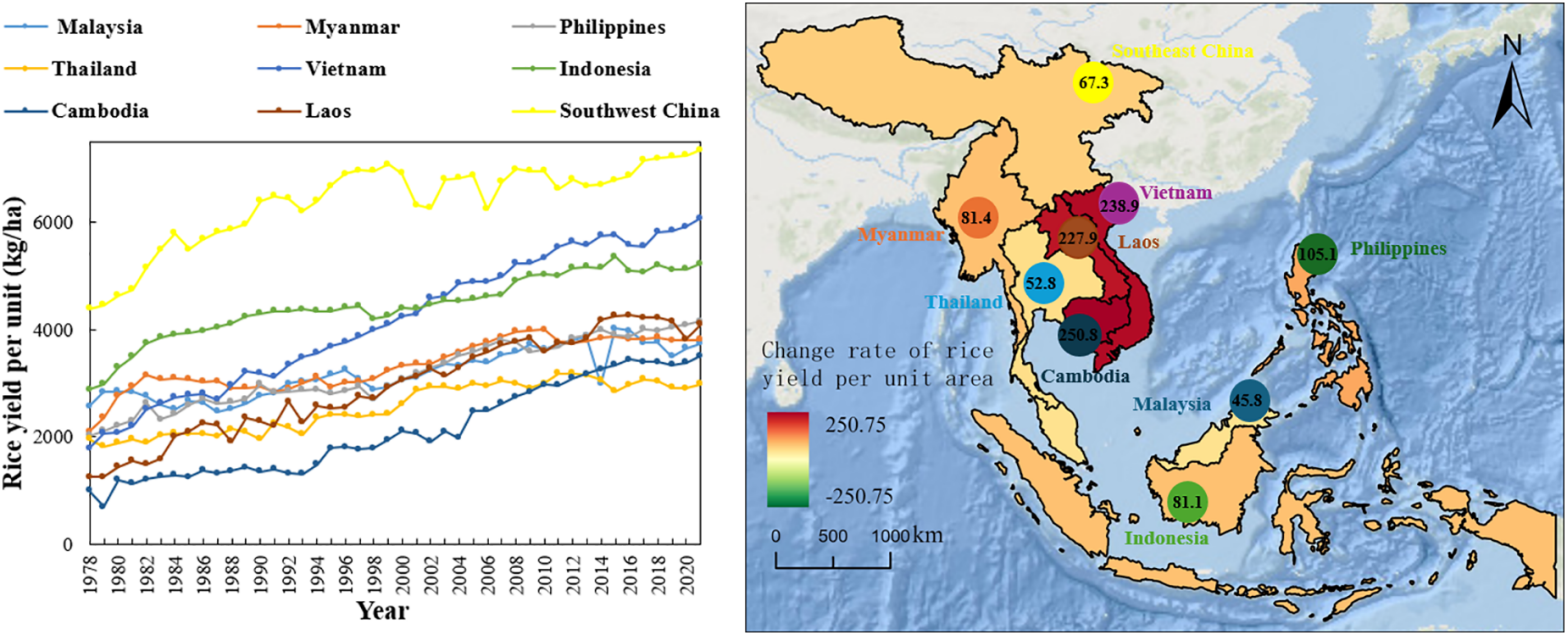
Variation in rice yield per unit area over the years (1978–2021) in southwest China and Southeast Asia.

The results illustrated that rice yields in Southwest China and the main rice-producing countries in Southeast Asia had a significant upward trend from 1978 to 2021 (**Figure 3**), with annual average increases of 6.87 kg ha^-1^ and 419.22 kg ha^-1^, respectively. Three countries, Cambodia, the Lao People’s Democratic Republic, and Vietnam, experienced significant increases in rice yields, by a factor of 2.51, 2.28, and 2.39, respectively. Among them, Vietnam’s rice yield per unit area is in a leading position among the major rice-producing countries in Southeast Asia. In 2021, the rice yield per unit area was 6073.96 kg ha^-1^, accounting for 18.07% of the total rice yield per unit area of the major rice-producing countries in Southeast Asia during the same period. Rice yields in Indonesia showed a fluctuating upward trend, increasing from 2886.23 kg ha^-1^ in 1978 to 5226.31 kg ha^-1^ in 2021, with an average annual increase of 54.42 kg ha^-1^. Rice yields in the Philippines, Myanmar, Malaysia, and Thailand showed a trend of slow growth year after year, with annual average increases of 49.5 kg ha^-1^, 39.76 kg ha^-1^, 27.34 kg ha^-1^ and 23.99 kg ha^-1^, respectively. During the same period, rice yields in the southwestern region of China increased from 4,391.19 kg ha^-1^ in 1978 to 7,345.53 kg ha^-1^ in 2021, an increase of 0.67 times. Rice yield in Southwest China has been increasing from 1978 to 2021, with an average annual increase of 68.7 kg ha^-1^, although its increase is the seventh largest in the main rice producing countries in Southeast Asia, but the annual rice yield in Southwest China is much higher than that in the main rice producing countries in Southeast Asia, and only in 2021, rice yield in Southwest China accounted for 21.86% of that in the main rice producing countries in Southeast Asia.

In summary, rice yields increased significantly in the main rice-producing countries of Southeast Asia compared to the southwestern China. Rice yields are growing rapidly in Vietnam, where government assistance in optimizing rice cultivation techniques and standardizing rice production has led to significant growth in yields. Rice is one of the major food crops in various countries and regions in Southeast Asia. Since the 1990s, countries have been actively introducing and breeding high-yielding hybrid rice and have been vigorously promoting it, which has led to a significant increase in rice yields.

#### 3.1.3 Comparative analysis on total rice production

**Figure 4:**
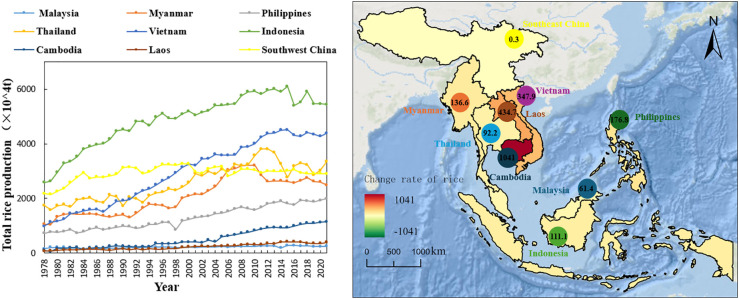
Change rate of total rice planting yield in southwest China and Southeast Asia.

The total rice production in Southwest China and the main rice-producing countries in Southeast Asia shows a general upward trend from 1978 to 2021 (**Figure 4**). Of these, Cambodia’s total rice production has grown most significantly, from 1 million tonnes in 1978 to 11.41 million tonnes in 2021, an average annual increase of 242,093 tonnes. This is followed by a more significant upward trend in total rice production in Laos and Vietnam, with average annual growth rates of 8.09% and 10.11%, respectively, and total rice production in 2021 of 3.87 million tonnes and 43.85 million tonnes, respectively. Indonesia’s total rice production is firmly ranked as the top rice producing country in Southeast Asia, growing from 25.77 million tonnes in 1978 to 54.41 million tonnes in 2021, an increase of 28.64 million tonnes. The total rice production of the Philippines, Myanmar and Thailand as a whole shows an increasing trend, with an average annual increase of 4.11, 3.17 and 2.14%, and the total rice production in 2021 was 19.96 million tonnes, 24.91 million tonnes and 33.58 million tonnes, respectively. Malaysia’s total rice production has remained stable at 12.33% of that of the major rice-producing countries in Southeast Asia, increasing from 1,498,000 tonnes in 1978 to 2,418,100 tonnes in 2021, with an average annual growth rate of 21,398 tonnes. During the same period, total rice production in the Southwest China region showed a fluctuating upward trend, increasing from 21,780,700 tonnes in 1978 to 28,961,000 tonnes in 2021, with the average annual increase in total rice production accounting for only 5.96% of that of the main rice-producing countries in Southeast Asia.

In summary, the total rice production in the southwestern region of China and the main rice-producing countries in Southeast Asia both show positive growth trends from 1978 to 2021. The further development of hybrid rice in China has led to an increase in total rice production in the southwestern part of the country, but the geography of the southwestern part of the country has led to a relatively small increase in production. Compared to the southwestern part of the country, the efficiency of rice yield in Cambodia has been improving while the advantage of production scale has been increasing, resulting in a significant increase in total rice production.

#### 3.1.4 Analysis of yield contribution and dominant types

The difference in rice contribution between Southwest China and the main rice producing countries in Southeast Asia during the same period (**Table 4**). In the first period (1978-2000), the area contribution rate of Southwest China was 0.094, indicating that the area sown to rice in Southwest China decreased in that period, but during the same period, the yield contribution rate of Southwest China was as high as 1.149, and the efficiency of its rice yields was much higher than that of the main rice-producing countries in Southeast Asia. In the first phase in the Southwest of the country was clearly dominated by the single-yield. The main rice-producing countries in Southeast Asia with yield contributions greater than 2.00 are Myanmar, the Philippines, Thailand, Vietnam and Laos. Rice production in all five of these countries was yield-dominant in 1978-2000. The yield contributions of Malaysia, Indonesia and Cambodia were 0.970, 1.636 and 1.235, respectively, indicating that the above three countries belong to the area and yield mutual domination type. From the first stage (1978-2000) to the second stage (2001-2021), the contribution rate of rice yield in Southwest China decreased from 12.223 to -1.396, indicating a decline in the rice production efficiency in Southwest China. However, the second stage was affected by the reduction of sowing area, which led to the transformation of Southwest China from a yield-dominant type to an area-dominant type. Malaysia and Indonesia shifted from mutual dominance (0.970, 1.636) to area dominance (-4.924, -2.019). The contribution of yield in the first stage was 3.840 and 5.939 in the Philippines and Laos, respectively, and the contribution of yield in the second stage was 1.669 and 1.188, respectively, indicating that rice production in the Philippines and Laos is transitioning from yield-dominated to area- and yield-dominated interactions. Thailand and Vietnam shifted from yield-dominant (3.143, 3.390) to area-dominant (0.355, -11.460). Rice production in Myanmar has been yield-dominant from 1978-2021, ranging from 2.269 in the first stage to 6.832 in the second stage. This suggests that the main reason for the increase in Myanmar’s total rice production is the improvement in its rice yield technology. However, rice production in Cambodia was dominated by area and yield interactions at both stages.

**Table 4:** Contribution of harvested area, yields and production in Southwest China and major rice producing countries in Southeast Asia, 1978–2021.

Country	Contribution of sown area		Yield contribution per unit of production		Rate of contribution to production	
	1978-2000	2001-2021	1978-2000	2001-2021	1978-2000	2001-2021
Malaysia	0.462	-0.269	0.449	1.324	0.970	-4.924
Myanmar	0.26	0.126	0.589	0.858	2.269	6.832
Philippines	0.187	0.336	0.717	0.561	3.840	1.669
Thailand	0.224	0.717	0.702	0.255	3.143	0.355
Vietnam	0.174	-0.1	0.589	1.141	3.390	-11.460
Indonesia	0.316	-1.207	0.518	2.438	1.636	-2.019
Cambodia	0.298	0.36	0.369	0.389	1.235	1.081
Laos	0.119	0.4	0.708	0.475	5.939	1.188
Southwest China	0.094	4.603	1.149	-6.424	12.223	-1.396

Compared with the southwestern region of China, Indonesia, Vietnam, Thailand, and Myanmar all have an index of scale advantage in rice production that is greater than 0, and the trend is increasing. In contrast, the scale dominance index of rice production in Laos, Cambodia, and Malaysia are all less than 0. In the first period (1978-2000), the Philippine rice production scale dominance index was -0.275, but in the second period (2001-2021) the rice production scale dominance index shifted to 0.024. It shows that rice sowing in the Philippines is expanding and compares favorably with the scale of rice production in the southwestern part of the country. At the same time, only Myanmar’s rice productivity advantage index is greater than 0 and shows a decreasing trend, while all other major rice-producing countries in Southeast Asia have a rice productivity index less than 0 and all show a decreasing trend.

**Figure 5:**
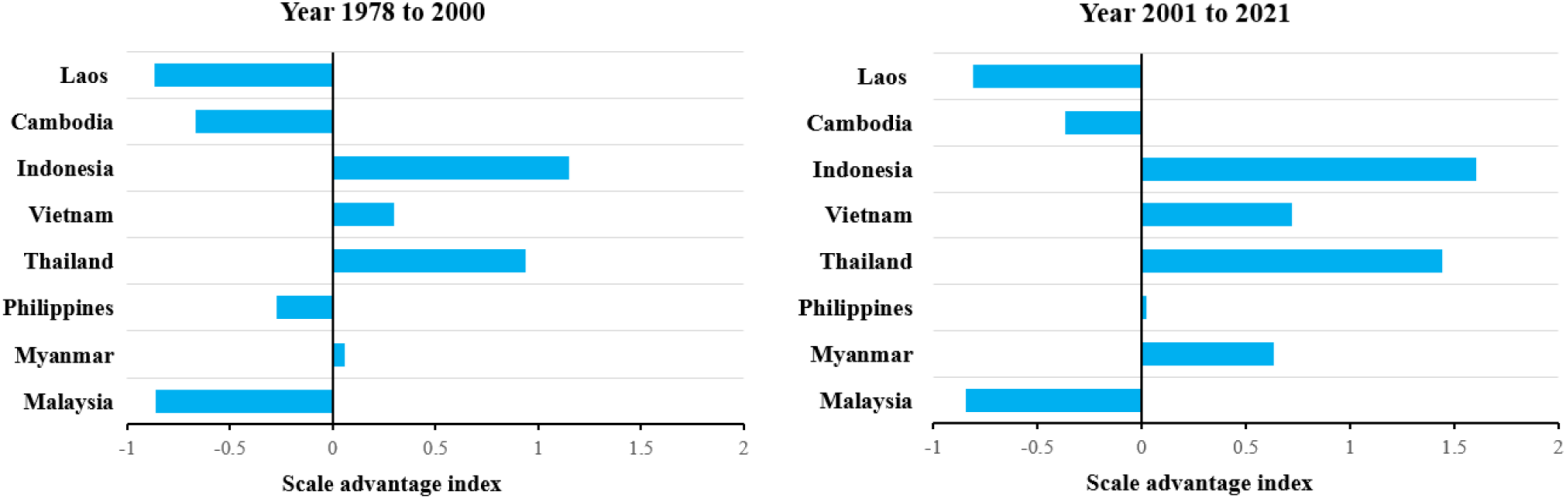
Scale advantage analysis of southwest China and Southeast Asia major rice producing countries from 1978–2000 and 2000–2021.

**Figure 6:**
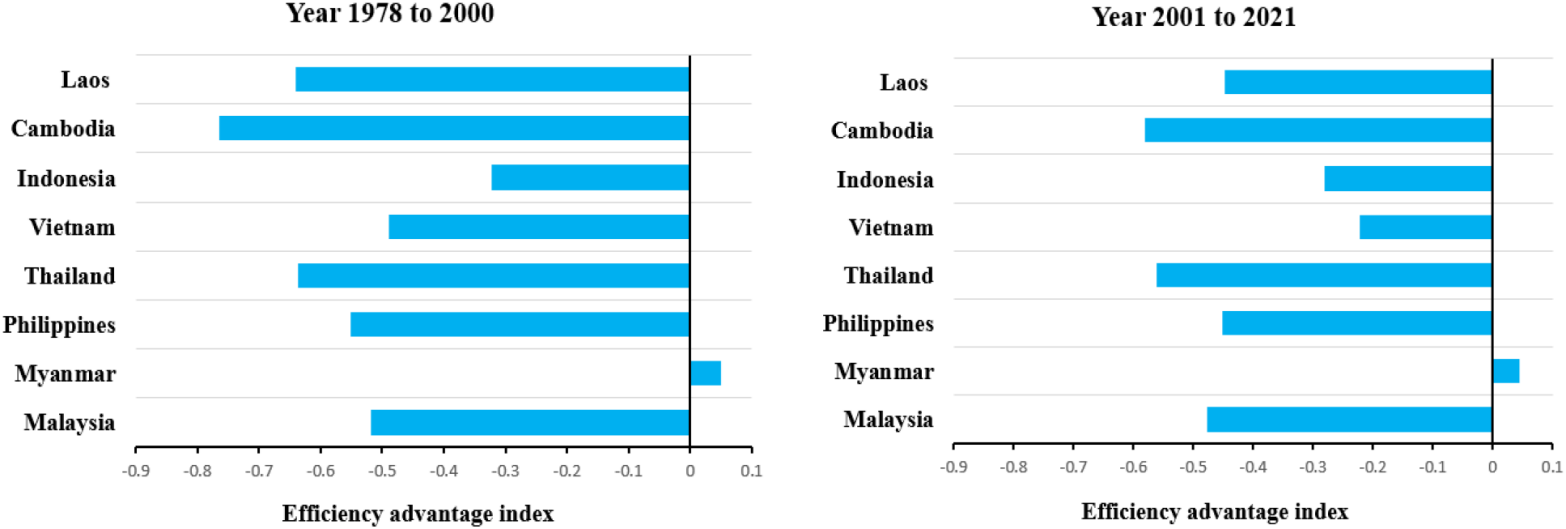
Efficiency advantage analysis of southwest China and Southeast Asia major rice producing countries from 1978–2000 and 2000–2021.

In summary, compared with the southwestern region of the country, the Philippines has achieved an index shift in the scale advantage of rice production due to the late development of hybrid rice in the first phase (**Figure 5**), which was in a state of experimentation, and the increase in the area of rice harvested after 2001 with the strong support of the national government. Myanmar has an overall advantage over the efficiency of rice production in the southwest China (**Figure 6**). However, there is a small decreasing trend of 8.82% in the index of superiority of rice productivity in Myanmar from the first phase to the second phase. This is mainly due to the increased efficiency of rice production in the southwest China.

### 3.2 Analysis of import and export

We examined the current situation of rice import and export trade between China’s southwestern region and the main rice-producing countries in Southeast Asia from 2011 to 2021.

#### 3.2.1 Analysis of the current situation of import and export

**Figure 7:**
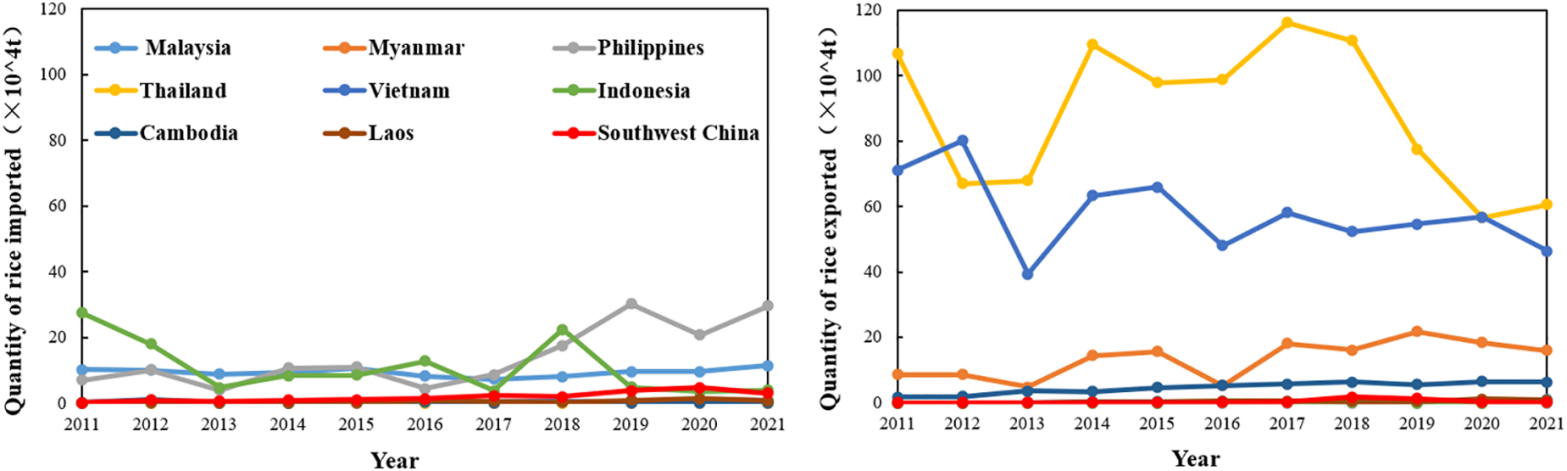
Change characteristics of rice trade volume between southwest China and Southeast Asia from 2011 to 2021.

Philippine rice imports have been increasing since 2011, and in 2021, Philippine rice imports accounted for about 62.88% of the main rice-producing countries in Southeast Asia (**Figure 7**). From 2011 to 2021, rice imports in southwest China increased and were net importers. The fluctuating increase in total rice production in southwest China is facing an imbalance in production and high consumer demand for supply and demand shortages. Based on this, southwestern China is actively protecting arable land, stabilizing the total amount of rice production, and using corresponding measures to guarantee the basic food supply in southwest China. During the same period, in terms of rice export volume, Thailand and Vietnam, as major rice exporting countries, had an average annual rice export volume of 881300 tons and 578300 tons respectively, accounting for 53.49% and 35.10% of the rice export volume of Southeast Asian rice producing countries, respectively. However, rice exports from Thailand and Vietnam have shown a fluctuating downward trend since 2011. Exports from Malaysia, the Philippines, Indonesia, Cambodia, Laos, and southwest China are at a lower level.

Overall, China’s southwestern region and the Philippines, Malaysia, and Indonesia maintain a relatively stable trend of rice imports, as the main rice importing countries or regions. While, Thailand, Vietnam, Myanmar, and Cambodia have shown a trend in rice exports and are the main rice exporting countries. Rice export areas are all located in the Central and Southern Peninsula, geographically concentrated, with the Southwest of China’s rice trade having significant geographic advantages.

#### 3.2.2 Country-by-country modelling of import and export

##### 3.2.2.1 Market share analysis

**Figure 8:**
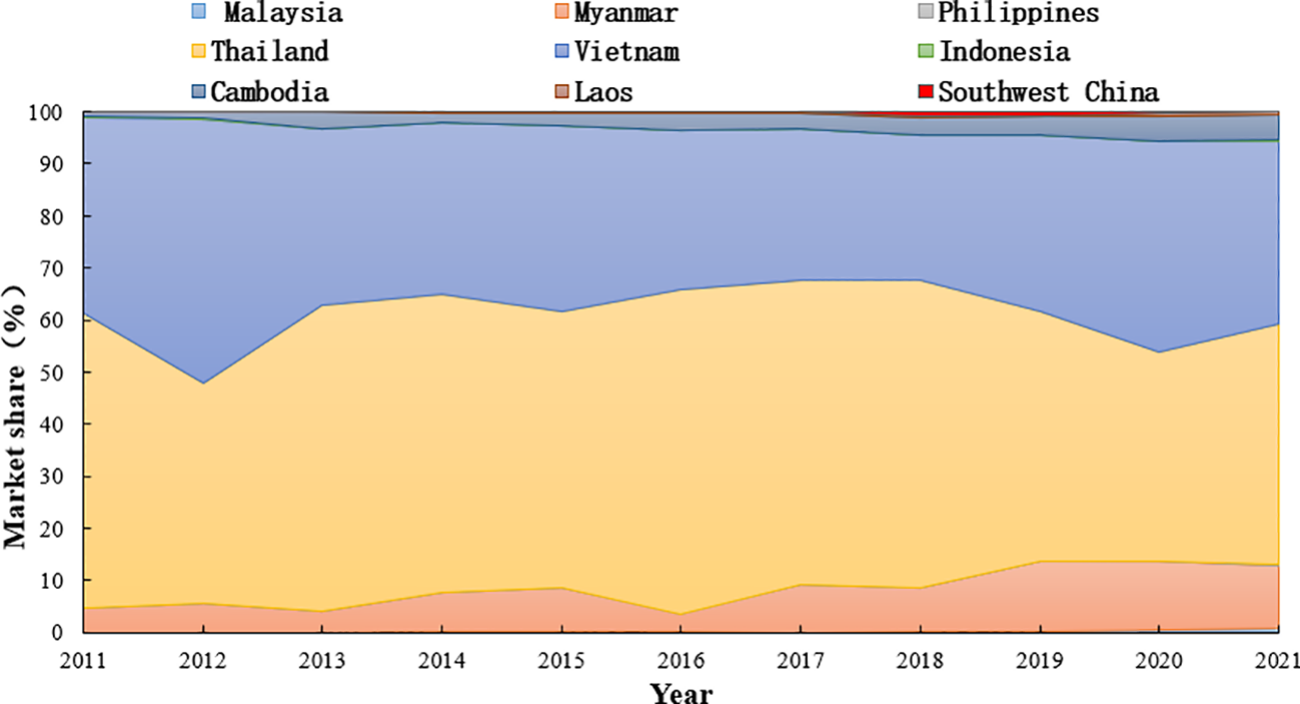
Change of export proportion of southwest China and major rice-producing countries in Southeast Asia from 2011–2021.

Studying the rice market share of southwestern China and the main rice-producing countries in Southeast Asia from 2011 to 2021 showed that the market share of Southwest China is 0.2% (**Figure 8**). This might be due to the insufficient supply of total rice production in southwest China and the low level of exports. Among the major rice-producing countries in Southeast Asia, Thailand, Vietnam, and Myanmar have significantly increased market share. Thailand accounted for 52.93% of the rice market, occupying an important position among rice exporters in Southeast Asia and being one of the main countries importing rice in southwest China. Vietnam and Myanmar also accounted for 35.3% and 8.2%, respectively, of the rice market in major rice-producing countries in Southeast Asia. The small increase in the market share of Cambodian rice is due to the small production of Cambodian rice in the early period of time, which is not enough to support the development of rice exports, but in recent years the rapid development of Cambodian rice and the continuous increase in the production of rice, which has gradually expanded the market for rice exports. However, Cambodia’s limited arable land resources prevent a significant increase in exports, and the market share is forecast to stabilize in the coming years.

##### 3.2.2.2 Analysis of net imports

From 2011-2021, the value of net rice imports in China’s southwestern region are greater than 0, and year by year an upward trend, from 0.12 million tonnes in 2011 to 30.28 million tonnes in 2021, an increase of 251.33 times, which indicates that China’s southwestern region is a rice importing region (**Table 5**). And among the main rice-producing countries in Southeast Asia, the net imports of Malaysia, the Philippines and Indonesia are all greater than 0 and stable, the same as in the Southwest of China, all of which belong to the import-type countries. Of these, the Philippines has seen a significant increase in the value of its net imports since 2018, with a marked increase in its dependence on foreign trade. During the same period, net imports were less than zero in Thailand, Vietnam, Myanmar and Cambodia, suggesting that rice trade in these four countries is dominated by exports. Among them, Thailand and Vietnam have large and stable export volumes and are highly competitive in exports among the major rice-producing countries in Southeast Asia. Apart from that, the net import of Laos is small, fluctuating between positive and negative, indicating that rice production in Laos is basically at the stage of self-sufficiency, and its demand for foreign trade in rice is small.

**Table 5:** Net imports of Southwest and Southeast Asian rice major producing countries.

Year	Southwest China	Malaysia	Philippines	Indonesia	Thailand	Vietnam	Myanmar	Cambodia	Laos
2011	0.12	103.04	70.61	274.45	-1066.06	-710.96	-86.43	-15.47	
2012	8.28	100.45	100.80	180.12	-667.86	-798.33	-82.10	-8.07	
2013	5.01	88.89	39.71	47.10	-676.65	-393.28	-46.41	-32.02	
2014	8.08	90.55	107.65	84.32	-1094.41	-630.37	-144.68	-32.34	-1.16
2015	10.16	101.46	109.48	86.06	-975.48	-654.86	-155.99	-39.82	-2.13
2016	13.60	77.64	44.61	128.15	-985.55	-474.65	-52.88	-50.33	4.52
2017	20.99	73.12	87.32	37.78	-1159.66	-575.26	-181.07	-56.90	0.83
2018	3.25	78.84	176.41	225.05	-1105.81	-517.35	-161.36	-61.06	-1.27
2019	26.51	94.96	302.94	49.17	-772.61	-540.54	-216.81	-53.21	5.85
2020	45.89	91.20	207.86	35.59	-561.99	-557.12	-184.31	-63.62	3.05
2021	30.28	104.53	296.61	40.45	-603.99	-455.49	-159.75	-62.56	0.10

In summary, based on net rice imports, four countries, Thailand, Vietnam, Myanmar and Cambodia, are exporting countries. Of these, Thailand and Vietnam have large and competitive exports. Malaysia, the Philippines, Indonesia and the southwest China are importing countries or regions. And net imports from China and the Philippines are on an upward trend, with growing import demand and a strong dependence on foreign trade in rice.

##### 3.2.2.3 Trade competitiveness analysis

**Figure 9:**
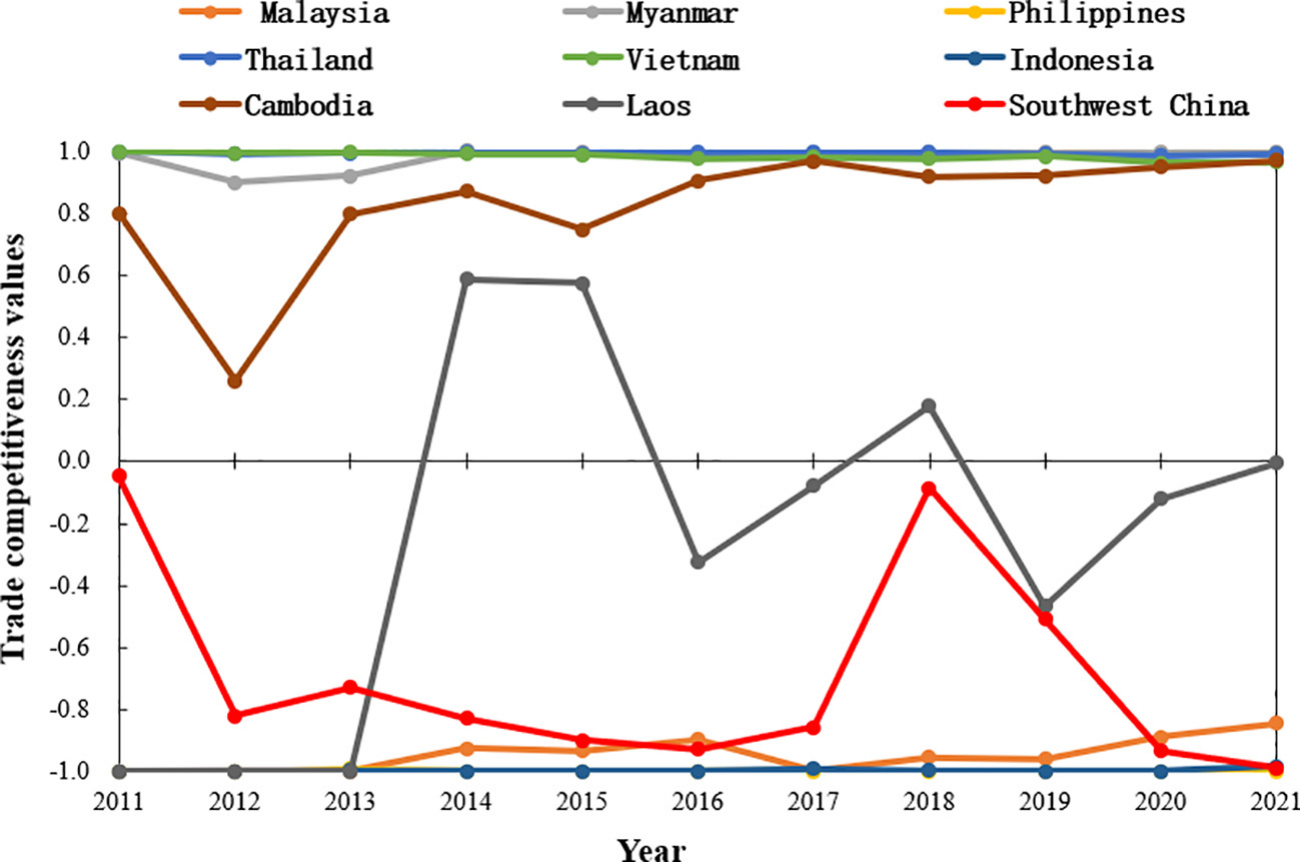
Comparison of changes in the trade competitiveness index (TC) between the Southwest and major rice-producing countries in Southeast Asia, 2011–2021.

Thailand, Myanmar, and Vietnam have the strongest rice trade competitiveness, with annual average TC indices of 0.994, 0.981, and 0.983, respectively, in 2011-2021, indicating that their rice trade has strong international competitiveness (**Figure 9**). This is followed by Cambodia, whose TC index is on an upward trend, growing from 0.8 in 2011 to 0.969 in 2021, and which also has a strong potential to compete in rice trade among the major rice-producing countries in Southeast Asia. The TC index of Laos fluctuates markedly, suggesting that Laos has unstable rice trade competition among the major rice-producing countries in Southeast Asia. Indonesia, Malaysia and the Philippines have annual average TC indices of -0.996, -0.944, and -0.999, respectively, indicating that their rice trade competitiveness is weak. The overall trend of rice TC index in the Southwest of the China is declining, from -0.046 in 2011 to -0.987 in 2021, indicating that its rice trade competitiveness is gradually decreasing.

### 3.3 Analysis of China’s Southwest Region’s rice import and trade potential

#### 3.3.1 Empirical results and analyses of trade attraction models

Through regression analysis of the data related to rice trade between Southwest China and the main rice-producing countries in Southeast Asia, the results were obtained (**Table 6**). The $R^{2}$ fit value is 0.439, which indicates that the model has a fair fit and can be interpreted. Among them, China’s southwestern rice imports of Southeast Asian rice-producing countries in each variable significant case, the main rice-producing countries in Southeast Asia crop sown area, per capita GDP in the southwest region to produce a positive and significant effect. The coefficients indicate that, all other things being equal, a one-unit increase in crop area in the major rice-producing countries of Southeast Asia will drive 0.01% of rice imports in the southwest China. This suggests that rice supply is an important factor influencing rice imports in the southwest China. Each 1% increase in per capita GDP in the southwest China will lead to a 0.006% increase in rice imports in the southwest China. Each unit increase in the GDP of the main rice-producing countries in Southeast Asia will lead to a 3.85% reduction in rice imports in the southwestern part of the country. The geographical distance between the main rice-producing countries of Southeast Asia and the southwestern China, as well as the distance of the economic system, negatively affects rice imports from the southwestern region. When geographical distance increases by 1%, rice imports decrease by 10.34%, indicating that transport costs remain the main constraint on rice imports. For every 1-unit increase in economic regime distance, rice imports decreased by 0.042%, suggesting that economic regime distance has a greater negative impact on rice imports in the southwest China.

**Table 6:** Regression results of the gravity model of rice trade between China’s southwestern region and the main rice producing countries in Southeast Asia.

Variant	Regression coefficient	T	P
$lndist_{ij}$	-10.340	-3.00	0.004
$S_{j}$	0.010	3.67	0.000
$lnGDP\_per_{it}$	0.0006	3.25	0.002
$regulation_{ijt}$	-0.042	-1.74	0.086
$GDP_{ijt}$	3.851	4.98	0.000
R^2^		0.439	
Adj-R^2^		0.399	

#### 3.3.2 Measurement of empirical results and analytical potential of trade gravity models

According to the value of potential change (**Table 7**), the Philippines belongs to the type with great potential, indicating that there is still a lot of room for development of rice imports to the Philippines from the southwestern China. Myanmar, Thailand, Vietnam, Cambodia and Laos are in the potential development category. It shows that the southwestern China has better prospects for the development of rice trade with these five countries, and there is still room for progress, and there is a need to take full advantage of trade and break down trade barriers. The rice trade relationship between China and Cambodia is strong and stable, with both sides showing positive developments in terms of trade size, policy agreements, economic impacts and future outlook, which has facilitated the development of rice trade with the southwestern China. With the deepening of global economic integration and regional cooperation, as well as the promotion of China’s Belt and Road Initiative, China’s rice trade cooperation with Myanmar, Thailand and Vietnam is expected to be further strengthened. The above shows that the development of rice trade between China and the major rice-producing countries in Southeast Asia has been on a favorable trend. In recent years, as a result of the signing of the Regional Comprehensive Economic Partnership (RCEP) trade agreement, the deepening of economic cooperation within the region has provided a better platform for rice trade in the southwestern China and achieved the integration of trade in agricultural products. Therefore, China’s southwestern region should make full use of the policy dividend to strengthen rice trade cooperation and guarantee food security, in the light of the specific situation of the main rice-producing countries in Southeast Asia. Among the major rice-producing countries in Southeast Asia, Thailand, Vietnam, Myanmar and Cambodia are export-oriented countries with a focus on exports.

**Table 7:** Changes in trade potential of Southwest China’s rice imports from Southeast Asian rice major producing countries, 2015–2021.

Year	Myanmar	Philippines	Thailand	Vietnam	Cambodia	Laos
2015	0.9614		1.0122	1.0188	1.0493	0.9708
2016	1.0225	0.6875	0.9685	1.0030	0.9665	0.9728
2017	1.0825		0.9489	1.0699	1.0412	0.9760
2018	1.1079	0.6715	1.0187	1.0105	1.0707	0.9779
2019	1.1387		1.0491	0.9862	1.0348	0.9763
2020	0.9358		1.0031	0.9424	1.0412	0.9907
2021	0.9332		1.0517	1.0486	0.9859	0.9291
均值	1.0260	0.6795	1.0075	1.0113	1.0271	0.9705

Through the above comparison can be seen, China’s southwestern region of rice international trade competitiveness is weak, mainly dependent on rice import trade, for the import-type region, and foreign rice trade imports increased year by year, increasing the demand for foreign rice imports. Among the major rice-producing countries in Southeast Asia, Thailand, Vietnam, Myanmar and Cambodia are export-oriented countries with a focus on exports. Although Cambodia’s export volume is relatively small compared to the other three countries, it is increasing year by year and has great trade potential and good trade prospects. Therefore, Thailand, Vietnam, Myanmar, and Cambodia can be the co-operative countries for our future rice import trade. Indonesia, Malaysia, and the Philippines, which are importing countries similar to ours may become competitors for our rice imports in the future. We need to take measures to achieve synergistic development of import and export trade between southwest China and other countries.

## 4 Discussion

Our study elaborated that from 1978-2021 China’s southwest rice sowing area had a downward trend (**Figure 2**), therefore it is important to improve the rice yield technology that has the potential to fluctuate upward trend of rice production. For instance, ratoon rice plantations can be adopted to achieve sustainable crop production, high-yielding, and high-efficiency rice production. China has almost 3.4 million hectares of land suitable for ratoon rice cultivation however only 1.24 million hectares were utilized will 2020 (Xu et al., 2021). The largest sowing area of ratoon rice is in southwest China and is nearly 40% of the current total land area devoted to ratoon rice (Jiang et al., 2021).

In addition, compared to China, the rice-producing countries in Southeast Asia have higher rice yields (**Figure 3**), and total rice production (**Figure 4**). However, analyzing rice production elaborates that the total output of rice production in China is unstable, and there is a downward trend in the second phase (2001-2021), and the main influencing factors are the reduction of rice sowing due to urbanization and industrialization (Long, 2014). Due to the serious fragmentation of arable land in southwest China, the rapid development of the economy and the increasing area of land for construction have made it impossible for arable land to be produced in a highly concentrated manner, and the contradiction between the rapidly developing population and the limited arable land resources has led to a further decrease in the area sown to rice in the southwestern part of China.

The total amount of rice production in China shows a trend of “north-south flush”, although the southern part of China is still the main rice-producing area in China, however, the center of gravity of China’s food production is constantly moving northward (**Figure 4**). It shows that the main rice-producing areas in China are developing at different speeds, and the high rice development areas have led to the low rice development areas, making the total rice output in China an overall upward trend. In addition, the current stage of food supply capacity in the Southwest of the country is rising amidst fluctuations (Jin and Zhong, 2022). To stabilize food security in the southwestern region of China, there is a trend of increasing demand for rice imports in the future.

The rapid growth of rice in the main rice-producing countries of Southeast Asia in the second phase (2001-2021) indicates that the economic development of most countries in the first phase (1978-2000) lagged, and the immaturity of rice-growing technology did not allow for large-scale agricultural development, and there was a high potential for land reclamation (**Table 3**). The spread of China’s hybrid rice technology (Ma and Yuan, 2015) in Southeast Asia has led to an increase in the total volume of rice production in the main rice-producing countries of Southeast Asia. It is noteworthy that rice production in Thailand, Myanmar, Vietnam, Laos, and Cambodia, which are concentrated in the central and southern peninsulas (Jaskuła et al., 2021), is dominant among the major rice-producing countries in Southeast Asia.

The relative concentration of arable land provides sufficient driving force for rice production, and the convenient geographical location provides better opportunities for rice trade, enabling the above five countries to rapidly capture the rice trade market. As for Indonesia, Malaysia, and the Philippines, due to the impact of extreme weather in recent years and the backwardness of their infrastructural conditions and cultivation techniques, resulting in lower total rice production than other major rice-producing countries in Southeast Asia, greater dependence on rice imports and a low share of the rice market, the lack of competitiveness of rice exports, and the inability to open up the rice trade market, which led to a very low level of exports of rice in the above three countries. Furthermore, China has less exports and limited contribution in market share compared to Thailand, this indicates that China should improve rice exports and rice growing strategies to increase its contribution in market share. In addition, China-Indochina Peninsula Economic Corridor (CIPEC), OBOR, and BRI policies have also improved the trade and economic development in these countries (Bashir et al., 2021).

On 15 November 2020, the country acceded to the RCEP. Currently, ASEAN is an important trade partner of China in RCEP, and the major rice-producing countries in Southeast Asia have all joined the agreement. The Southwest of China is an important “gateway” for ASEAN’s trade exchanges with mainland of China in the areas of economic trade, logistics, and transport, providing greater convenience for rice trade between the Southwest of China and the major rice-exporting countries in Southeast Asia (Chen and Zhao, 2023).

At present, as the global climate continues to warm, affected by rising temperatures and decreasing precipitation, the frequency, degree, and extent of droughts in the Southwest are all showing an increasing trend. The frequency, degree, and extent of droughts in the Southwest of China are all showing an increasing trend, and the warm-drying climate is becoming more pronounced. High-temperature heat damage shifts from mild to moderate and severe ratings. The lack of scientific and reasonable irrigation and drainage management and irrigation and drainage technology systems, resulting in unstable rice yield, quality, and so on. These problems are not conducive to guaranteeing food security in southwest China. Therefore, there is a need to ensure that the red line of food security is not crossed in a way that deepens foreign cooperation in rice agriculture and develops the food industry in the Southwest of the country in a high-quality manner (Srinivasan et al., 2023).

In summary, the Southwest of China has a high demand for rice. The rice products provided by the main rice-producing countries in Southeast Asia are not only of high yield and quality but also of a wide variety to meet the needs of different consumers. In terms of policy, the Chinese Government has been encouraging the diversification of trade in agricultural products, and through the establishment of stable trade relations with the main rice-producing countries in Southeast Asia, it can promote the balanced development of the import and export of agricultural products in the Southwest of China. In terms of complementarity, there is a strong trade complementarity between the main rice-producing countries in Southeast Asia and the Southwest of China in terms of rice and other agricultural products. The Southwest of China can import high-quality rice products from these countries while exporting competitive agricultural products or manufactured goods to them, achieving mutual benefits. The farming community can understand the benefits of rice cultivation, production and trade in southeast Asian countries and focus on meeting the global food demand and international trades. In addition, rice traders and policy makers can develop future trade cooperation with Southeast Asian rice producing countries to benefit global trade.

## 5 Conclusion

### 5.1 Increased agricultural inputs and systems to ensure production and regional food security

The Chinese government should increase the policy inclination towards the Southwest by formulating and implementing policies that encourage farmers to plant rice through financial subsidies, credit support, and other means to stabilize rice production. In addition, ensure that agricultural production is adequately safeguarded through the implementation of a series of effective institutional measures to maintain and enhance the security and stability of food supply in the entire southwest region. At the same time, the government should also implement relevant land policies to ensure the rational use and protection of land resources, especially paddy fields, and encourage the transfer of land and large-scale operations. In the development process, adhere to the red line of arable land, reduce the fragmentation of arable land, increase the degree of intensification of rice production, and strengthen the support and promotion of agricultural science and technology to improve crop yields and quality. Furthermore, emphasis should be placed on the development and improvement of relevant infrastructure to provide better production environments and living conditions for cultivators.

### 5.2 Deepening the reform of the rice industry chain, increasing the added value of rice, and expanding the rice trade market in Southeast Asia

Promoting rice production growth and focusing on the rice industry is important to learn from their institutional reforms related to the development of the rice industry. Changing the unitary pattern of rice trade can effectively enhance the competitiveness and added value of rice products by improving production efficiency, innovating processing technologies, and increasing marketing strategies. At the same time, it should actively explore and develop the rice trade market in Southeast Asia and take advantage of the integration of resources within the region to achieve the international development of the rice industry. This will help the Southwest break down international trade barriers, broaden trade channels, and promote the sustainable and healthy development of the rice industry.

### 5.3 Establishing a high-standard rice free trade zone to promote international market integration

The governments of southwest China and Southeast Asian countries should adopt a proactive policy of cooperation by simplifying trade procedures, preferential taxation, improving infrastructure, and other related policies. Encourage and support the construction of a free trade zone for rice and promote the liberalization and facilitation of rice trade between southwest China and Southeast Asia. On the other hand, by investing in and improving transport, warehousing, logistics, and other infrastructures. The establishment of modern “ports”, and logistics centers to ensure the timely transport of rice, can further reduce trade costs and improve the rice trade efficiency. Additionally, establish a monitoring and improved trade assessment mechanism to provide feedback on the operation of the rice free trade zone and make timely adjustments to the relevant policies to ensure the smooth and efficient operation of the rice free trade zone.

### 5.4 Strengthening the trade and cultivating new trade growth points

Against the backdrop of a globalized economy, the RCEP provides us with a unique opportunity to strengthen trade links between Southwest China and Southeast Asian countries, particularly the major rice-producing countries. This not only promotes the international circulation of agricultural products but also helps to cultivate new growth points in foreign trade and enhance the competitiveness of China’s agricultural trade. Through an in-depth understanding and knowledge of the provisions of the text of the agreement, the Government should act proactively to take advantage of policy dividends, such as tariff reductions and exemptions, as a means of facilitating the expansion of trade in rice. Taking RCEP as an opportunity to give full play to its role in promoting rice trade will help build a more open and inclusive new pattern of international agricultural trade. By strengthening cooperation and exchange, we can work together to create a more prosperous rice industry and make new contributions to food security in the Southwest of China.

In conclusion, our study helps policymakers, rice industrialists, and agricultural professionals, and farmers to understand market dynamics, optimize resource allocation, and develop strategies to enhance food security and economic growth in the region. The study lacks trade among the countries of crops other than rice as well as potential export with countries other than South China. It can be studied in future for more beneficial outcomes.

## Data Availability

The original contributions presented in the study are included in the article/supplementary material. Further inquiries can be directed to the corresponding authors.
